# Highly sensitive voltammetric determination of Pb²⁺ and Cd²⁺ ions using a carbon paste electrode modified with Mn₀.₅Zn₀.₅Fe₂O₄ spinel ferrite nanoparticles

**DOI:** 10.1038/s41598-026-52676-4

**Published:** 2026-05-20

**Authors:** Asmaa A. Khodari, Ahmed A. Shamroukh, Ahmed R. Tawfik, Hassan M. A. Salman, Hytham F. Assaf

**Affiliations:** Chemistry Department, Faculty of Science, Qena University, Qena, 83523 Egypt

**Keywords:** Mn₀.₅Zn₀.₅Fe₂O₄ nanoparticles, Carbon paste electrode, Adsorptive stripping voltammetry, Lead and cadmium detection, Heavy metal monitoring, Electrochemical sensor., Chemistry, Environmental sciences, Materials science, Nanoscience and technology

## Abstract

A novel electrochemical sensing platform based on a Mn_0.5_Zn_0.5_Fe_2_O_4_ nanoparticles-modified carbon paste electrode (MnZnFe NPs/CPE) was developed for the simultaneous voltammetric determination of trace Cd²^+^ and Pb²^+^ ions. MnZnFe NPs were synthesized via co-precipitation method and characterized by FTIR, XRD, SEM&EDX, and TEM analyses, confirming the formation of a well-crystallized spinel ferrite structure with nanoscale morphology. The incorporation of MnZnFe NPs significantly enhanced the electrocatalytic activity of the carbon paste matrix, providing improved adsorption capacity and electron-transfer kinetics for metal ions. Adsorptive stripping differential pulse voltammetry (ASDPV) was employed for analytical measurements under optimized conditions (pH 5, deposition potential − 1.2 V, deposition time 120 s). The modified electrode exhibited well-defined and separated oxidation peaks for both Cd²^+^ and Pb²^+^, achieving low detection limits 0.21 nM and 0.03 nM for Cd²^+^ and Pb²^+^ respectively, wide linear ranges 0.80 nM – 28.0 µM and 0.12 nM– 30.0 µM for Cd²^+^ and Pb²^+^ respectively, and excellent reproducibility (RSDs of 0.42% (Cd²^+^) and 1.39% (Pb²^+^)). The sensor also demonstrated high selectivity toward Cd²⁺ and Pb²⁺, with negligible interference from common coexisting ions, and showed good stability over repeated use. The developed MnZnFe NPs/CPE provides a simple, low-cost, and efficient approach for monitoring toxic heavy metals in environmental water samples.

**Supplementary Information:**

The online version contains supplementary material available at 10.1038/s41598-026-52676-4.

## Introduction

The rapid industrialization and extensive use of heavy metals in industrial, agricultural, and domestic activities have resulted in serious contamination of natural water resources. Heavy metal ions are of particular concern because they are non-biodegradable, tend to accumulate in biological systems, and pose severe risks to human health and ecosystems even at very low concentrations. Among these toxic pollutants, lead (Pb²^+^) and cadmium (Cd²^+^) are considered priority hazardous metals by environmental protection agencies worldwide due to their high toxicity and widespread occurrence in water, soil, and food chains^1,2^. Lead exposure is known to cause irreversible damage to the nervous system, especially in children, and is associated with cognitive impairment, anemia, kidney dysfunction, and cardiovascular diseases. Cadmium, on the other hand, is a highly toxic and carcinogenic metal that can accumulate in the kidneys and liver, leading to renal failure, osteoporosis, and pulmonary disorders. Because the permissible limits of Pb²^+^ and Cd²^+^ in drinking water are extremely low (in the µg L^−^¹ range), the development of sensitive, selective, and reliable analytical methods for their determination is critically important for environmental monitoring and public health protection^3,4^.

Traditional analytical techniques such as atomic absorption spectroscopy (AAS)^5^, inductively coupled plasma mass spectrometry (ICP-MS)^6^, and inductively coupled plasma optical emission spectroscopy (ICP-OES) are widely used for heavy metal analysis because of their excellent sensitivity and accuracy^7^. However, these methods typically require expensive instrumentation (commonly USD 20,000–300,000 depending on configuration), dedicated laboratory facilities, complex sample preparation, and skilled operators, which limit their routine on-site applicability. Analytical and electroanalytical methods play a central role in modern environmental and biological monitoring because they enable accurate identification and quantification of trace chemical species in complex matrices. In environmental systems, such methods are widely used for monitoring toxic heavy metals, pesticides, pharmaceuticals, and emerging contaminants in water, soil, and food samples. In biological and biomedical analysis, they are equally important for the determination of drugs, metabolites, biomolecules, and clinically relevant ions at very low concentrations^8–14^. Stripping voltammetry—has gained increasing attention due to their operational simplicity, low cost, rapid response, and excellent sensitivity^15,16^. Portable potentiostats are commercially available at costs often below USD 1,000–10,000, while analysis times are commonly within 2–10 min per sample.

Moreover, stripping voltammetric methods can achieve trace-level detection limits in the low µg L⁻¹ to ng L⁻¹ range (approximately 10⁻⁹–10⁻¹² M for many metal ions), making them highly competitive with established spectrometric methods. Their compact instrumentation, low reagent consumption, and compatibility with field-deployable systems further make them highly suitable for in situ environmental monitoring.

Carbon paste electrodes (CPEs) are attractive platforms for electrochemical sensing due to their wide potential window, low background current, chemical inertness, ease of fabrication, and renewable surface. Moreover, CPEs can be readily modified with functional materials to improve their electrochemical performance. However, conventional CPEs also present certain limitations, including moderate mechanical stability, possible binder swelling or drying during prolonged measurements, susceptibility to surface fouling, and batch-to-batch variability caused by manual paste preparation. In recent years, the incorporation of nanostructured materials into carbon paste matrices has proven to be an efficient strategy to enhance the electroactive surface area, facilitate charge transfer, and improve the accumulation efficiency of heavy metal ions at the electrode surface^17,18^.

Spinel ferrite nanoparticles with the general formula MFe₂O₄ (where M = Mn²⁺, Zn²⁺, Co²⁺, Ni²⁺, etc.) have emerged as promising electrode modifiers owing to their nanoscale dimensions (typically 10–100 nm), specific surface areas commonly in the range of 30–150 m² g⁻¹ depending on synthesis route, high chemical stability, semiconducting conductivity typically between 10⁻⁶ and 10⁻² S cm⁻¹, and rich redox activity arising from mixed-valence Fe²⁺/Fe³⁺ and transition-metal cation couples. Their electrical and catalytic properties can be tuned through cation substitution, enabling improved adsorption capacity and faster interfacial electron transfer. Recent studies have highlighted the sensing performance of spinel ferrites in electrochemical applications, demonstrating enhanced analytical sensitivity and lower detection limits for trace analytes^19–21^. Among these materials, manganese–zinc ferrites (MnₓZn₁₋ₓFe₂O₄) exhibit superior electrochemical behavior due to the synergistic effect between Mn²⁺ and Zn²⁺ ions within the spinel lattice. Manganese contributes to enhanced redox activity and charge-transfer capability, while zinc improves structural stability and provides additional adsorption sites for metal ions, making Mn–Zn ferrites highly attractive for electrochemical sensing applications^22–27^.

In this study, a novel Mn₀.₅Zn₀.₅Fe₂O₄-modified carbon paste electrode (MnZnFe NPs/CPE) was developed for the sensitive voltammetric determination of Pb²⁺ and Cd²⁺ ions. The synthesized spinel ferrite nanoparticles were characterized using standard physicochemical techniques, and their integration into the carbon paste matrix was evaluated for electroanalytical performance using adsorptive stripping differential pulse voltammetry (ASDPV). The proposed sensor exhibited excellent sensitivity, low detection limits, and reliable performance in the presence of common interfering ions. This work highlights the potential of MnZnFe NPs as an efficient, low-cost, and easily prepared electrode modifier for trace heavy metal monitoring in environmental water samples.

## Experimental

### Materials and reagents

All chemicals used in this study were of analytical grade and were employed without further purification. Graphite powder (spectroscopic grade, 1–2 μm, Sigma-Aldrich, USA) and paraffin wax (high purity, Sigma-Aldrich, USA) were used to prepare the bare carbon paste electrode. Lead (II) nitrate and cadmium (II) nitrate tetrahydrate (≥ 99%, Sigma-Aldrich, USA) were used as standard sources of Pb²⁺ and Cd²⁺ ions. Stock solutions of each metal ion were prepared daily in deionized water (18.2 MΩ·cm) and diluted to obtain working solutions of the desired concentrations. Acetate buffer (0.1 mol L⁻¹, pH 5) was prepared from glacial acetic acid and sodium acetate (Sigma-Aldrich, USA) and used as the supporting electrolyte for all voltammetric measurements.

### Synthesis of MnZnFe NPs

MnZnFe NPs was synthesized by a conventional chemical coprecipitation route. Stoichiometric amounts of Fe(NO_3_)_3_·9H_2_O, Zn(NO_3_)_2_·6H_2_O, and Mn(NO_3_)_2_·4H_2_O (≥ 98%, Sigma-Aldrich, USA) were weighed to satisfy the cationic ratio Mn: Zn: Fe = 0.5:0.5:2, then dissolved in 50 mL of deionized water to obtain a clear homogeneous mixed-metal solution. Then, 0.5 g of citric acid was introduced into the solution as a capping agent, and the mixture was vigorously stirred. A 2 M NaOH solution was prepared separately and added dropwise to the vigorously stirred metal nitrate solution at room temperature until the pH reached about 10–11, promoting complete coprecipitation of the corresponding metal hydroxides. The suspension was further stirred and heated at 70–80 °C for 1–2 h to age the precipitate and allow the nucleation and growth of the spinel precursor.

The resulting black precipitate was separated by filtration, repeatedly washed with deionized water followed by ethanol to remove residual ions and by-products, and then dried in an oven at 80–100 °C overnight. The dried powder was gently ground in an agate mortar to break soft agglomerates, then calcined in a muffle furnace at 1200 °C for 2 h to induce crystallization of the spinel MnZnFe NPs phase^28^, after which the product was cooled to room temperature in the furnace and stored in a desiccator until characterization and further use.​.

### Instrumentations and apparatus

FTIR spectra of the synthesized MnZnFe NPs were recorded in the range 4000–400 cm⁻¹ using the KBr pellet technique on an FTIR spectrometer (Shimadzu 8400 S, Japan). XRD pattern was obtained using a Bruker D-8 diffractometer (Germany) equipped with CuKα radiation (λ = 0.15406 nm). Data were collected over a 2θ range of 20–80° at a scan rate of 2° min⁻¹. The surface morphology and microstructural features of the nanocomposites were examined using scanning electron microscopy (JEOL JSM-IT200, Japan). Transmission electron microscopy (JEOL JEM-F200, Japan) operating at 200 kV with a spatial resolution of 0.23 nm was employed to further analyze particle size and assess detailed morphological characteristics of the prepared samples. Electrochemical measurements were performed using a computer-controlled potentiostat (Versa STAT4, USA) equipped with adsorptive stripping differential pulse voltammetry (ASDPV) capability. A conventional three-electrode system was employed, consisting of the MnZnFe NPs/CPE as the working electrode, an Ag/AgCl (3 M KCl) reference electrode, and a platinum wire counter electrode. MnZnFe spinel ferrite is a semiconducting material, while the effective conductivity of the fabricated paste electrode is primarily governed by the conductive graphite network. All experiments were conducted at room temperature (25 ± 2 °C).

### Preparation of the working electrodes

Bare Carbon Paste Electrode (BCPE) was prepared by homogenously mixing graphite powder with paraffin wax in a 75:25 (w/w) ratio. The mixture was lightly heated to ensure complete integration before being packed into the barrel of an insulin syringe, which functioned as the electrode body. Electrical contact was established using a cleaned copper wire firmly inserted into the packed paste to minimize interfacial contact resistance and ensure stable signal collection. On the other hand, the modified CPE with MnZnFe NPs (MnZnFe NPs/CPE) was fabricated using a similar procedure with an adjusted composition.

Briefly, MnZnFe NPs were first blended with graphite powder, and this composite was then combined with the paraffin wax binder. The final optimized composition consisted of 60% (w/w) graphite, 15% (w/w) MnZnFe NPs, and 25% (w/w) paraffin wax (see **Fig. ****S1**).

Accordingly, graphite (60%) was used to ensure sufficient electrical percolation and rapid charge transport within the paste matrix, whereas MnZnFe nanoparticles served mainly as electrocatalytic and adsorption-active modifiers. The resulting homogeneous paste was similarly packed into an electrode holder. Finally, for both electrodes, the surface of each freshly packed electrode was smoothed on clean paper to create a uniform, renewable disk. Prior to all electrochemical measurements, the electrodes were activated by applying cyclic voltammetry in a phosphate buffer solution (PBS, pH 5.0) between 0 and 1.0 V until a stable voltammetric response was obtained. This pretreatment the surface, increases electroactive surface area, and introduces oxygen-containing functional groups that improve conductivity and analytical response for analyte detection^29^.

### Real sample preparation

Water samples were sourced from two distinct origins: surface water from the Nile River at Qena, Egypt; and municipal tap water. Each sample underwent initial preparation by filtration through a membrane filter to remove particulate matter followed by dilution with 0.1 M acetate buffer (pH 5.0). Subsequently, each sample was analyzed for the presence of Cd^2+^ and Pb^2+^ ions using the proposed method at optimized conditions in conjunction with the standard addition method to ensure accuracy and account for potential matrix effects in the real water samples.

## Results and discussion

### Characterization of MnZnFe NPs

#### FTIR analysis

**Fig. 1:**
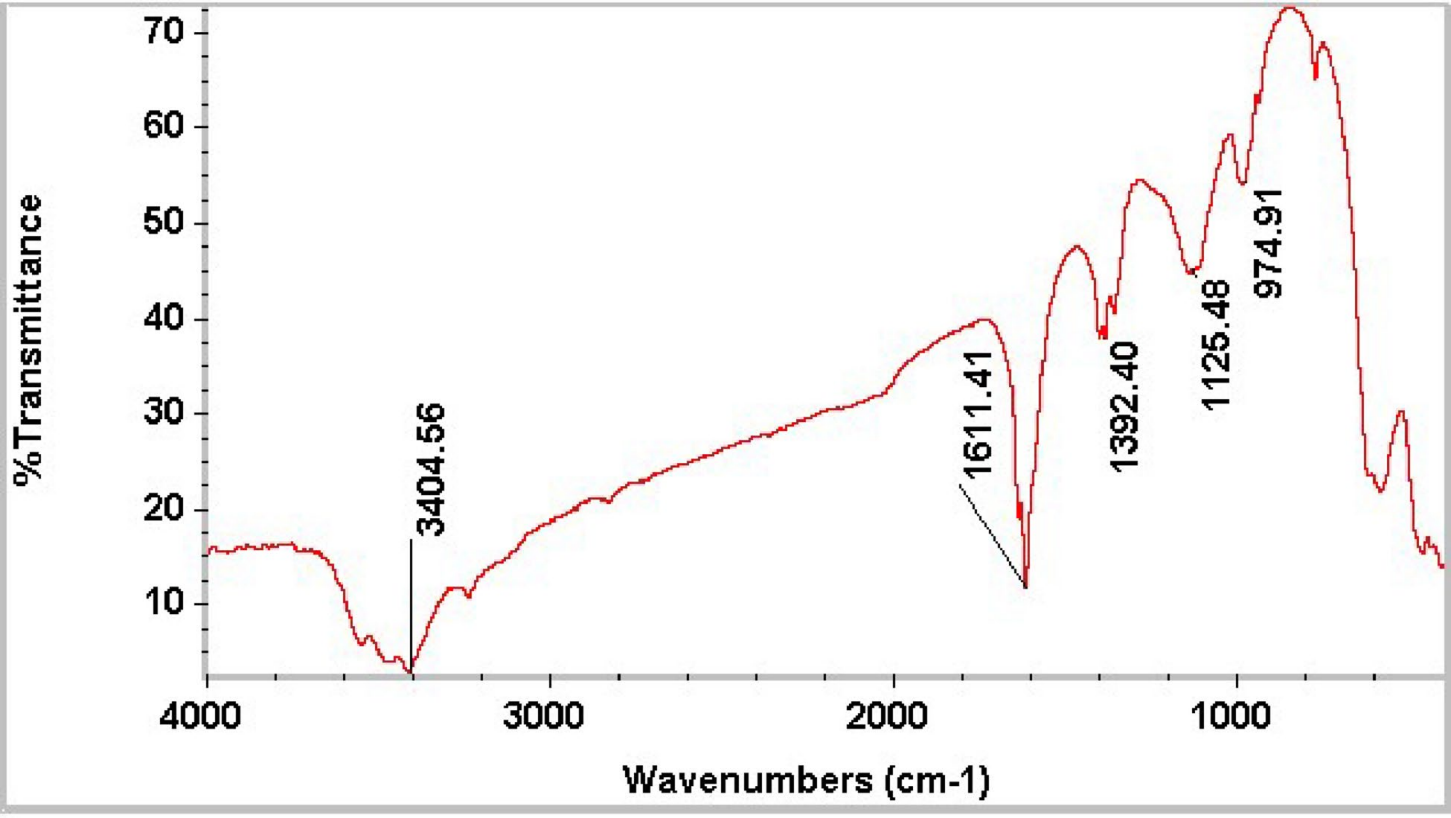
FTIR spectrum of the synthesized MnZnFe NPs.

The FTIR spectrum of MnZnFe NPs shows the characteristic vibrations of a spinel ferrite lattice together with surface hydroxyl and adsorbed species (Fig. 1). The broad band centered at about 3404 cm^− 1^ can be assigned to O–H stretching vibrations of adsorbed water molecules and surface hydroxyl groups hydrogen-bonded on the ferrite surface, while the weak feature in the 1600 cm^−^¹ region (1611 cm^−^¹) corresponds to the H–O–H bending mode of molecular water. Similar broad O–H stretching and H–O–H bending bands have been widely reported for Mn–Zn and other spinel ferrites prepared by wet-chemical or sol–gel routes.

The band at 1392 cm^−^¹ is usually attributed to residual nitrate species from precursor salts or to C–O stretching of adsorbed atmospheric CO_2_, which are commonly observed in ferrite powders dried at moderate temperatures^30,31^. The strong absorption features in the low-wavenumber region, particularly the bands around 1125 and 975 cm^−^¹, arise from metal–oxygen vibrations in the tetrahedral and octahedral sublattices of the spinel structure; in Mn–Zn ferrites, the higher-frequency band is associated mainly with stretching of metal–oxygen bonds at tetrahedral (A) sites, whereas the lower-frequency band is due to vibrations of oxygen atoms around octahedral (B)-site cations. The presence of these intense M–O bands in the 1100–900 cm^−^¹ region confirms the formation of the spinel MnZnFe NPs phase and indicates that the cations are incorporated into the ferrite lattice rather than remaining as separate oxide or hydroxide impurities^32^.​.

#### XRD analysis

**Fig. 2:**
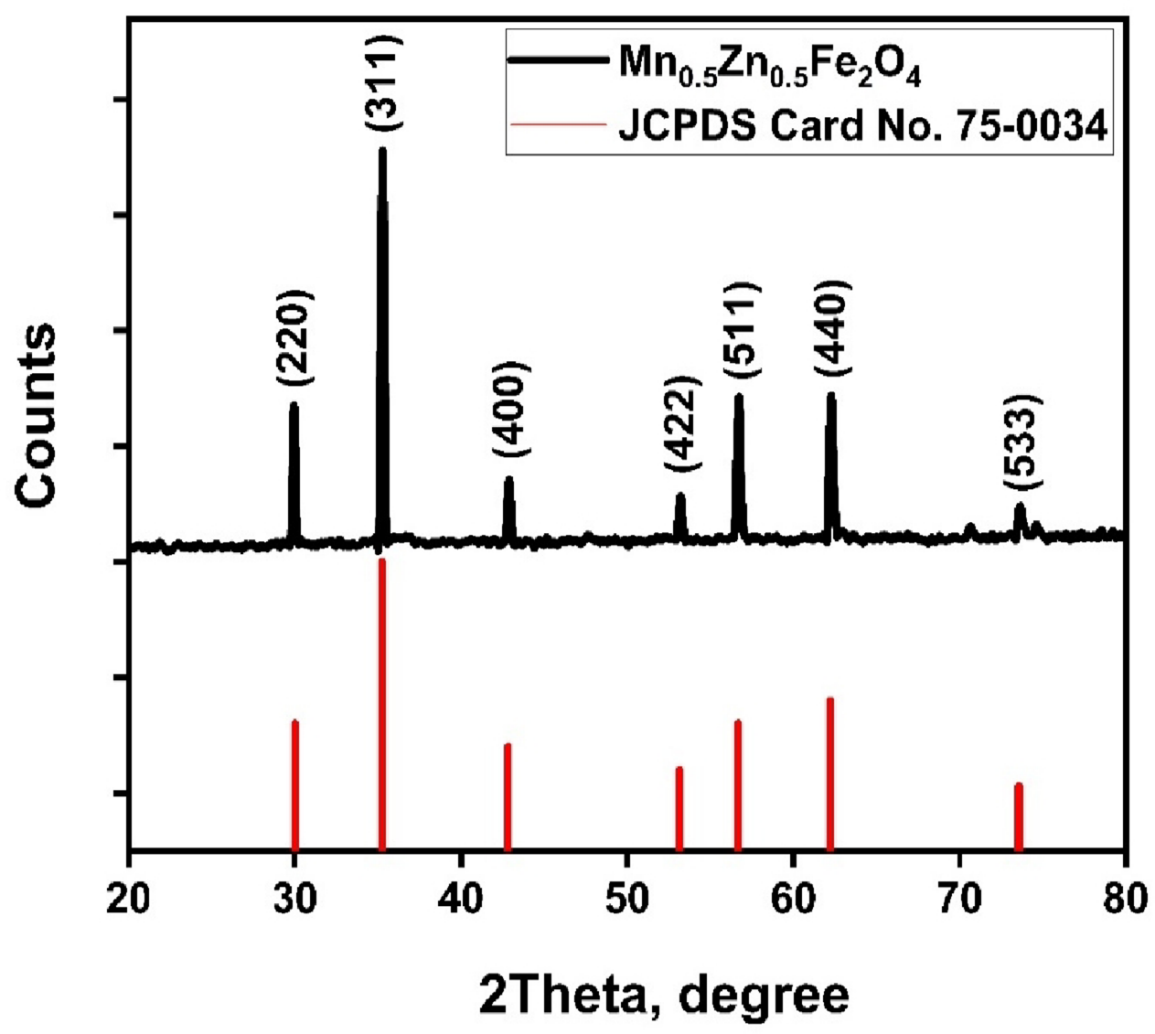
XRD pattern of the synthesized MnZnFe NPs.

The XRD pattern of the MnZnFe NPs sample reveals the formation of a well-defined cubic spinel structure, as evidenced by distinct peaks at 2θ values of approximately 29°, 35°, 42°, 53°, 56°, 62°, and 74°, which correspond to crystallographic planes (220), (311), (400), (422), (511), (440), and (533), respectively (Fig. 2). These reflections match closely with the reference standard (**JCPDS Card No. 75 − 0034**)^30,31^, confirming phase purity and the absence of secondary impurities such as ZnO, MnO, or α-Fe_2_O_3_ within the sample.

The (311) peak, being most intense, is characteristic of spinel ferrites and indicates that the sample’s cations are effectively distributed among tetrahedral and octahedral sites in the lattice, producing strong constructive interference. The sharpness and symmetry of all peaks further suggest nanocrystalline nature and high crystallinity, while the absence of peak broadening indicates minimal lattice strain or defects. The crystallite size of the sample was calculated from Scherer equation and found to be 59.3 nm. Overall, the XRD profile demonstrates that the synthesis procedures successfully yielded highly pure crystalline Mn-Zn ferrite nanoparticles suitable for further studies involving catalytic properties for voltammetric sensor developments.

#### SEM morphological analysis

**Fig. 3:**
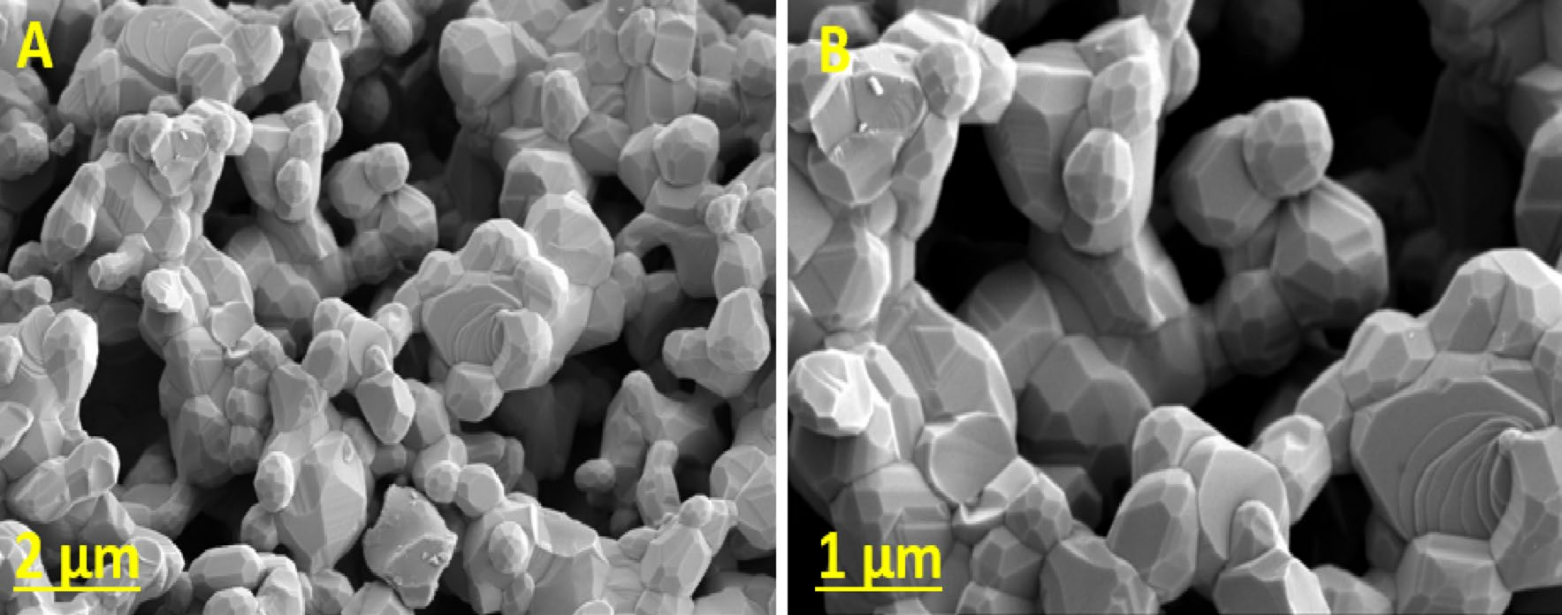
**A**,** B** SEM images of the synthesized MnZnFe NPs at different magnifications.

The SEM micrograph of MnZnFe NPs reveals a densely packed, three-dimensional architecture composed of agglomerated nanocrystals with well-defined faceted surfaces and irregular cauliflower-like aggregates (Fig. 3A, B). This morphology indicates a high degree of surface roughness and the presence of numerous interparticle voids and crevices, which are expected to increase the electroactive surface area and facilitate electrolyte penetration during voltammetric measurements.

The individual crystallites appear to grow radially, forming clustered structures with multiple exposed crystallographic planes that can provide a large population of adsorption and coordination sites for heavy metal ions. Such hierarchical architecture is expected to provide an enlarged effective surface area and multiple accessible active sites, which is beneficial for interfacial processes such as adsorption, mass transport through the porous network, and charge transfer in electrochemical applications. Overall, the SEM image confirms that the synthesized material possesses a robust, rough, and morphologically heterogeneous surface that is well suited for use as a functional modifier in electrode formulations and related surface-dependent technologies.

The energy-dispersive X-ray (EDX) spectrum of the synthesized MnZnFe NPs confirms the elemental composition and purity of the sample (**Fig. ****S1**). The spectrum exhibits prominent characteristic peaks corresponding to O, Mn, Fe, and Zn, verifying the successful formation of the mixed spinel ferrite structure. The strong peak observed at lower energy is attributed to oxygen (O K), while the distinct peaks appearing around ~ 0.6–1.5 keV and in the higher energy region (~ 6–9 keV) correspond to Mn, Fe, and Zn elements. In particular, Fe shows dominant peaks, indicating its major presence in the structure, which is consistent with the stoichiometry of MnZnFe NPs. The quantitative analysis reveals weight percentages of approximately 27.3% O, 11.5% Mn, 47.3% Fe, and 13.9% Zn, which are in close agreement with the expected elemental ratios. The absence of any impurity peaks in the spectrum confirms the high purity of the synthesized nanoparticles. The uniform presence of all constituent elements suggests homogeneous distribution within the sample. Overall, the EDX results validate the successful synthesis of phase-pure MnZnFe NPs with the desired chemical composition, supporting their suitability for applications in magnetic and electrochemical devices.

#### TEM analysis

**Fig. 4:**
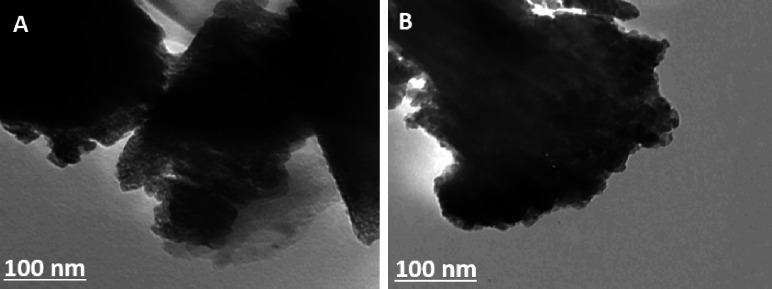
**A**,** B** TEM micrographs of the synthesized MnZnFe NPs.

The TEM images indicate that MnZnFe NPs sample consists of highly aggregated nanoparticles forming irregular, compact clusters with no long-range ordering (Fig. 4A, B). The contrast variation suggests assemblies of smaller primary crystallites fused into larger agglomerates, which is typical for spinel ferrite nanoparticles produced by wet-chemical routes due to strong magnetic dipole–dipole interactions and surface energy–driven aggregation. The darker regions correspond to thicker or overlapping particle domains, whereas the lighter fringes at the periphery of the clusters point to thinner edges composed of nanosized grains. This morphology is consistent with previous reports on Mn–Zn ferrite nanoparticles, where nearly spherical to slightly faceted crystallites coalesce into dense aggregates while retaining a nanoscale primary particle size beneficial for high surface area and enhanced functional performance.

### Electroactive surface area measurements

**Fig. 5:**
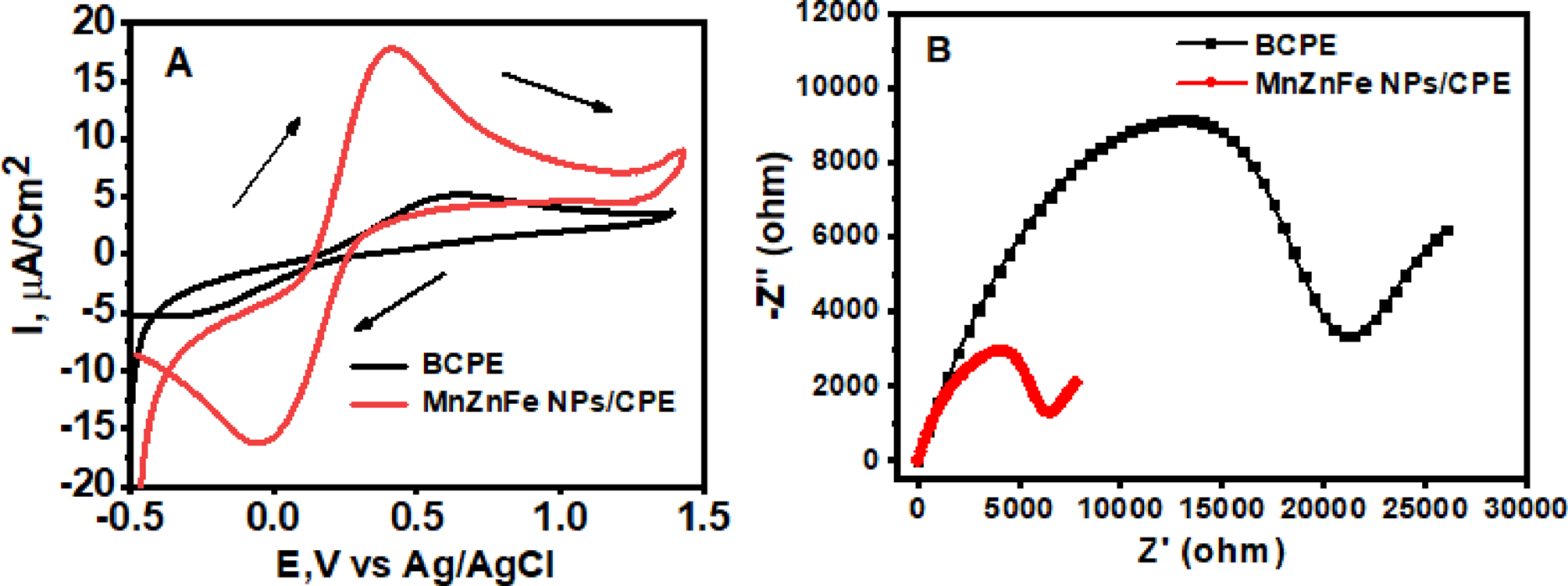
**(A)** CVs and EIS (**B**) of 1.0 mM of [Fe(CN)_6_]^3−/4−^ in KCl (0.1 M) solution at scan rate 50 mV/s; BCPE and MnZnFe NPs/CPE.

Figure 5A shows the cyclic voltammograms of 1.0 mM K₃[Fe(CN)₆]/K₄[Fe(CN)₆] in 0.1 M KCl recorded at BCPE and MnZnFe NPs/CPE at a scan rate of 50 mV s⁻¹. The voltammograms shown correspond to the third consecutive stabilization cycle after initial conditioning, with the scan initiated in the anodic direction. Prior to each experiment, the open-circuit potential was monitored for 60 s and only minor variations (< ± 10 mV) were observed, indicating stable electrode/electrolyte interfaces before polarization.

Both electrodes exhibited a redox response characteristic of the ferri/ferrocyanide probe; however, the relatively large peak-to-peak separations (ΔEp) indicate quasi-reversible rather than ideal reversible electron-transfer behavior under the present experimental conditions. The BCPE displayed a ΔEp value of 0.872 V, whereas the MnZnFe NPs/CPE showed a substantially lower ΔEp of 0.456 V. The larger separation observed at BCPE is attributed to sluggish electron-transfer kinetics, heterogeneous surface structure, and the partially insulating nature of the paraffin binder within the carbon paste matrix. In contrast, the marked decrease in ΔEp after incorporation of MnZnFe nanoparticles confirms accelerated interfacial electron transfer and reduced charge-transfer resistance. It should also be noted that a minor contribution from uncompensated solution resistance (iR drop) cannot be excluded, although the use of 0.1 M KCl as supporting electrolyte was intended to minimize this effect.

In addition to the reduced ΔEp, the anodic and cathodic peak current densities at MnZnFe NPs/CPE were significantly higher than those of BCPE, demonstrating enhanced electrochemical activity of the modified electrode. This improvement is attributed to the synergistic effects of MnZnFe nanoparticles, which increase surface roughness, provide additional electroactive sites, and facilitate charge transport through the graphite matrix.

The electroactive surface area (A) of each electrode was estimated from the Randles–Ševčík equation using the anodic peak current of the ferri/ferrocyanide couple:

$\:{\boldsymbol{I}}_{\boldsymbol{p}}=(2.69\times\:{10}^{5}){\hspace{0.17em}}{\boldsymbol{n}}^{1.5}\boldsymbol{A}{\boldsymbol{D}}_{\boldsymbol{R}}^{0.5}{\boldsymbol{\nu\:}}^{0.5}{\boldsymbol{C}}_{0}$where n is the number of transferred electrons (1), D is the diffusion coefficient of [Fe(CN)₆]³⁻/⁴⁻ (7.6 × 10⁻⁶ cm² s⁻¹), C is the bulk concentration (mol cm⁻³), and v is the scan rate (V/s)^33^. The calculated electroactive surface areas were 0.010 cm² for BCPE and 0.046 cm² for MnZnFe NPs/CPE, corresponding to a 4.6-fold increase after modification. This substantial enhancement explains the higher current response and supports the superior analytical performance of the modified electrode.

To further characterize the interfacial properties, electrochemical impedance spectroscopy (EIS) was carried out in the same redox probe solution (Fig. 5B). The Nyquist plot of BCPE exhibited a large semicircle in the high-frequency region, corresponding to a charge-transfer resistance (Rct) of approximately 21 kΩ, indicative of slow electron-transfer kinetics. After modification with MnZnFe nanoparticles, the semicircle diameter decreased markedly, giving an Rct value of approximately 6 kΩ. This pronounced reduction confirms that the MnZnFe nanostructure greatly facilitates electron transfer between the redox species and the electrode surface. The linear tail observed at lower frequencies for both electrodes is attributed to Warburg impedance, indicating diffusion-controlled mass transport following the charge-transfer step. Overall, the CV and EIS results consistently demonstrate that MnZnFe NPs modification significantly improves the electrochemical properties of the conventional carbon paste electrode by enlarging the active surface area and accelerating heterogeneous electron-transfer kinetics.

### Effect of MnZnFe NPs modification on the voltammetric detection of Cd²^+^ and Pb²^+^

**Fig. 6:**
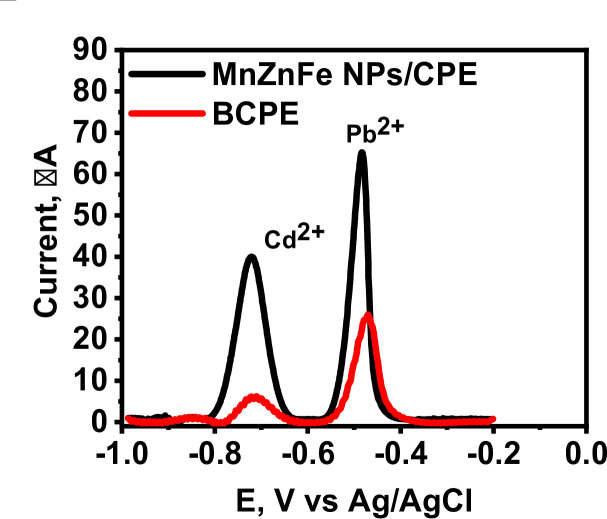
ASLSVs of the BCPE and MnZnFe NPs/CPE recorded in acetate buffer (pH 5.0) contains 1.0 µM Cd²^+^ and 2.0 µM Pb²^+^, deposition potential = −1.2 V, deposition time = 120 s.

The electrochemical behavior of the BCPE and MnZnFe NPs/CPE toward Cd²⁺ and Pb²⁺ was investigated by adsorptive stripping linear sweep voltammetry (ASLSV) in 0.1 M acetate buffer (pH 5.0) over the potential range of − 1.0 to 0.0 V versus Ag/AgCl. As shown in Fig. 6, the BCPE produced weak, broad, and poorly resolved anodic responses, whereas the MnZnFe NPs/CPE generated two sharp and well-separated stripping peaks with substantially enhanced current intensities, corresponding to the oxidation of electrodeposited Cd⁰ and Pb⁰ back to Cd²⁺ and Pb²⁺, respectively. The observed stripping peaks arise from the reoxidation of metals previously accumulated during the deposition step. Based on standard electrode potentials, the formal redox potentials of Cd²⁺/Cd and Pb²⁺/Pb versus Ag/AgCl are approximately − 0.61 V and − 0.33 V, respectively. The experimentally observed peak potentials were shifted relative to these thermodynamic values, which are expected under dynamic stripping voltammetric conditions due to nucleation and dissolution kinetics, adsorption phenomena, uncompensated resistance, and interfacial interactions with the modified electrode surface. Nevertheless, the relative peak order remained fully consistent with electrochemical thermodynamics, with Pb stripping at more positive potentials than Cd, thereby enabling reliable simultaneous determination.

The significant enhancement in stripping current at the MnZnFe NPs/CPE confirms the beneficial role of MnZnFe nanoparticles as an electrocatalytic modifier. This behavior can be attributed to several synergistic factors: (i) enlargement of the effective electroactive surface area, allowing greater accumulation of target metal ions; (ii) accelerated heterogeneous electron-transfer kinetics through redox-active Mn/Fe centers; (iii) improved electrical conductivity of the composite electrode through intimate contact with the graphite matrix; and (iv) favorable adsorption/preconcentration of Cd²⁺ and Pb²⁺ on the ferrite surface. In addition to signal amplification, the improved peak separation observed at the modified electrode demonstrates enhanced selectivity and reduced peak overlap, which is essential for simultaneous multicomponent analysis. The sharper voltammetric features also indicate faster stripping kinetics and more uniform deposition sites on the MnZnFe-functionalized surface^24–27^. Overall, these findings clearly demonstrate that incorporation of MnZnFe spinel ferrite nanoparticles markedly improves the analytical performance of conventional carbon paste electrodes, making the developed sensor highly suitable for sensitive and simultaneous electrochemical monitoring of trace Cd²⁺ and Pb²⁺ ions in environmental samples.

### Effect of deposition potential and deposition time

In stripping voltammetry, the deposition potential and deposition time are critical parameters, as they directly govern analytical sensitivity and the detection limit. These variables were systematically optimized using a 0.1 M acetate buffer at pH 5.0. As shown in **Fig. S3A**, the ASDPV peak currents for both target ions increased when the deposition potential was shifted from − 0.9 V to − 1.35 V but declined at more negative potentials. This decrease is attributed to hydrogen evolution at the electrode surface, which reduces its effective active area^34^. **Fig. S3B** demonstrates that the peak currents increased substantially as the deposition time was extended from 0 to 250 s. Beyond this period, the DPASV response plateaued, indicating that the electrode surface had reached saturation with the target metal ions^35^. Consequently, an optimal deposition potential of − 1.2 V and a deposition time of 120 s were selected for all subsequent experiments.

### Effect of supporting electrolyte pH

**Fig. 7:**
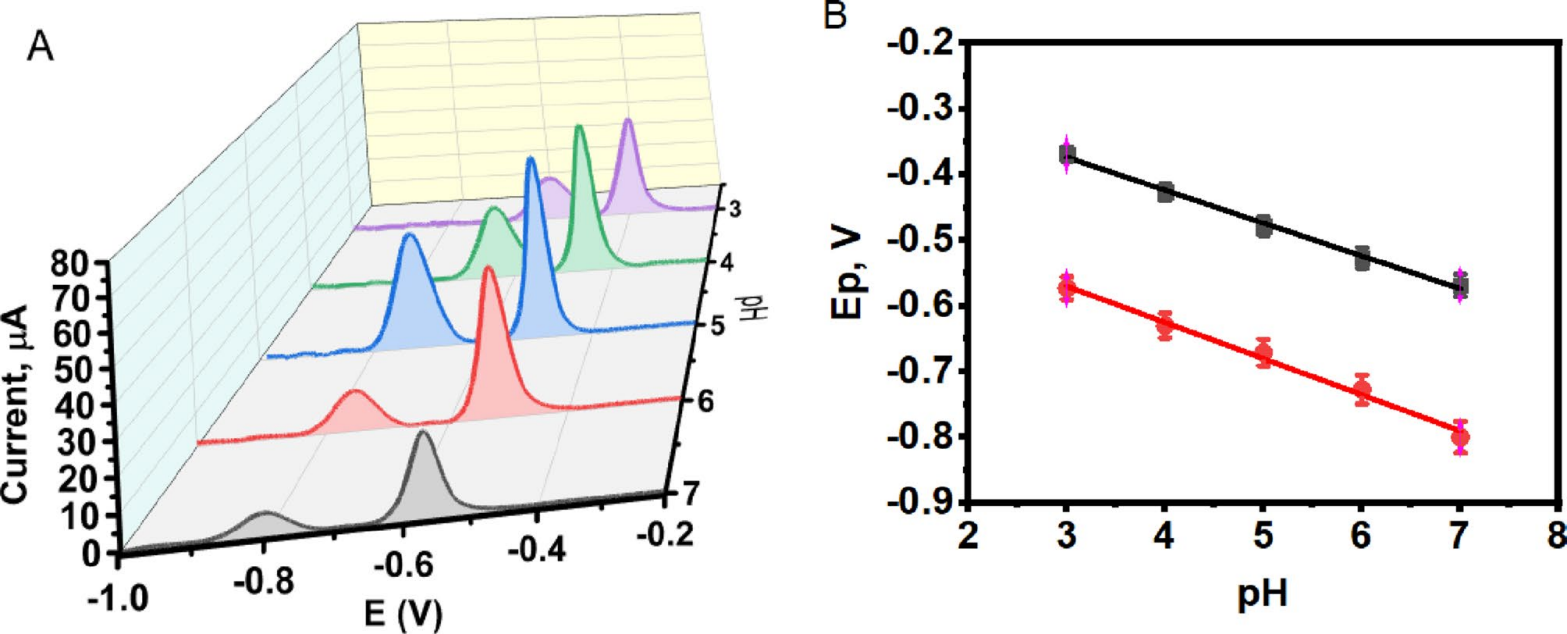
ASLSVs of 1.0 µM Cd²^+^ and 2.0 µM Pb²^+^ at MnZnFe NPs/CPE in 0.1 M acetate buffer (pH 5.0), deposition potential = −1.2 V, deposition time = 120 s: (A) scan rates 20–500 mV s¹; (B) Ip vs. v¹ᐟ²; (C) log Ip vs. log v.

The electrochemical oxidation behavior of Cd²^+^ and Pb²^+^ ions is highly dependent on the pH of the supporting electrolyte, as pH directly influences ion adsorption dynamics, proton availability, and charge transfer kinetics at the electrode solution interface factors that collectively determine sensor sensitivity and selectivity^36^. To systematically assess the pH effect, ASLSV was performed at MnZnFe NPs/CPE using 0.1 M acetate buffer across the pH range of 3.0–7.0. Upon increasing the pH value (**Fig. 7A**), the current response increases and then decreases gradually due to the formation of metal hydroxide which inhibited the accumulation of these metal ions as reported in literature^37^. Based on the combined evaluation of peak intensity, potential separation, and peak sharpness, pH 5.0 was selected as the optimal condition for the simultaneous electrochemical detection of Cd²^+^ and Pb²^+^ ions. Additionally, as pH increases, proton activity decreases and partial hydrolysis of Cd²⁺ and Pb²⁺ may occur, leading to changes in surface speciation and a displacement of the oxidation potential toward more negative values (**Fig. **7**B**). The linear relationships obtained were:

Ep(Cd²^+^) = − 0.2235 − 0.0501 pH (R² = 0.992).

Ep(Pb²^+^) = − 0.4047 − 0.0551 pH (R² = 0.991).

The slopes of approximately 50–55 mV/pH are close to the theoretical Nernstian value of 59 mV/pH at 25 °C, suggesting coupled proton/electron interfacial equilibria during the stripping process^38^.

**Figure 7 A** ASLSVs of 1.0 µM Cd²^+^ and 2.0 µM Pb²^+^ at MnZnFe NPs/CPE in 0.1 M acetate buffer at pH 3–7, scan rate 50 mV/s, deposition potential = −1.2 V, deposition time = 120 s, and (**B)** plot of oxidation peak potentials (Ep) versus pH for Cd²^+^ and Pb²^+^.

### Effect of scan rate

**Fig. 8:**
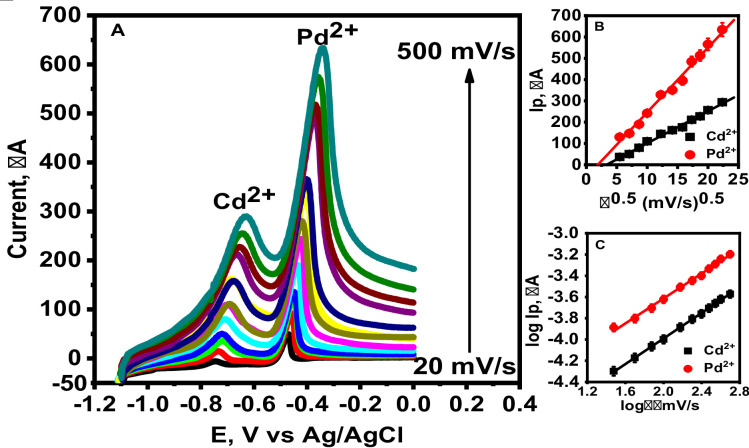
ASLSVs of 1.0 µM Cd²^+^ and 2.0 µM Pb²^+^ at MnZnFe NPs/CPE in 0.1 M acetate buffer (pH 5.0), deposition potential = −1.2 V, deposition time = 120 s: (A) scan rates 20–500 mV s¹; (B) Ip vs. v¹ᐟ²; (C) log Ip vs. log v.

The effect of scan rate on the anodic response of 1.0 µM Cd²^+^ and 2.0 Pb²^+^ µM was studied using ASLSV at MnZnFe NPs/CPE in 0.1 M acetate buffer (pH 5.0) at scan rates from 20 to 500 mV/s (Fig. 8A**).** Increasing the scan rate led to a rise in both peak currents and a positive shift in their potentials. A plot of peak current versus the square root of the scan rate (Fig. 8B) revealed linear relationships, described by the equations:

Ip (µA) = 10.11 v^0·5^ (mV/s) ^0·5 −^ 14.44 (R² = 0.994) for Cd²^+^

Ip (µA) = 18.95 v^0·5^ (mV/s) ^0·5^ − 41.9 (R² = 0.996) for Pb²^+^.

This behavior generally indicates diffusion-controlled mass transport, consistent with the Sevčík–Randles model^37^. However, it is important to note that the Sevčík–Randles equation is derived under ideal conditions assuming a reversible electron-transfer process, planar electrode geometry, semi-infinite linear diffusion, and negligible adsorption effects. In the present ASLSV system, these conditions are only partially satisfied due to the preconcentration step, surface confinement of metal ions on MnZnFe NPs/CPE, and possible kinetic limitations at higher scan rates. Therefore, slight deviations from perfect linearity are observed at elevated scan rates, which are commonly reported in nanoparticle-modified stripping systems^39^. Furthermore, log–log plots of peak current versus scan rate (Fig. 8C) yielded slopes near 0.5 for both ions:

log Ip (µA) = 0.58 log v (mV/s) – 4.788 (R² = 0.995) for Cd²^+^.

log Ip (µA) = 0.59 log v (mV/s) – 5.19 (R² = 0.994) for Pb²^+^.

These values confirm that diffusion remains the dominant process, although minor deviations from ideality suggest a mixed contribution from adsorption and kinetic effects. Such behavior is expected in modified carbon paste electrodes, where heterogeneous surface sites and nanostructured modifiers enhance adsorption and electron-transfer pathways. These observations are in line with recent studies reporting that Mn–Zn ferrites, significantly enhance electron-transfer efficiency and mass transport in CPEs. This enhanced performance is attributed to the synergistic effects of the ternary MnZnFe oxide structure, which provides abundant active sites, high ionic conductivity, and favorable intermetallic interactions that promote the effective adsorption and redox activity of heavy metal ions. Furthermore, the incorporation of Zn into the spinel ferrite framework enhances the electrochemical stability and charge transfer kinetics, leading to well-resolved and highly sensitive stripping peaks^24–27^. Therefore, Mn–Zn ferrites modification not only increases the electroactive surface area but also accelerates electron transfer, making the electrode highly suitable for the simultaneous voltammetric detection of Cd²^+^ and Pb²^+^ ions.

### Proposed electrochemical sensing mechanism of MnZnFe NPs/CPE toward Cd²^+^ and Pb²^+^ detection

The enhanced stripping voltammetric performance of the MnZnFe NPs/CPE toward Cd²⁺ and Pb²⁺ detection can be explained through a multistep synergistic sensing mechanism. First, the incorporation of MnZnFe spinel ferrite nanoparticles into the carbon paste matrix increases the effective electroactive surface area and introduces a rough, porous interface containing abundant adsorption-active sites. These surface hydroxyl groups and metal–oxygen centers promote preconcentration of Cd²⁺ and Pb²⁺ ions near the electrode surface through electrostatic attraction and surface complexation^21–24^. During the deposition step at negative potential (− 1.2 V), the accumulated Cd²⁺ and Pb²⁺ ions are electrochemically reduced to their metallic states according to:

Cd^2+^+2e^−^→Cd^0^.

Pb^2+^+2e^−^→Pb^0^.

The deposited metals nucleate on the conductive modified surface, where the graphite phase ensures rapid electron transport and the MnZnFe nanoparticles lower the interfacial charge-transfer resistance. During the subsequent anodic stripping scan, the deposited metals are re-oxidized back into solution, producing characteristic current peaks:

Cd^0^ → Cd^2+^+2e^−^.

Pb^0^ → Pb^2+^+2e^−^.

The higher stripping currents observed at MnZnFe NPs/CPE arise from the larger amount of preconcentrated metal, faster electron-transfer kinetics, and improved accessibility of active sites. The clear separation between Cd and Pb peaks results from differences in their thermodynamic oxidation potentials and surface interaction energies. Furthermore, the spinel lattice contributes additional catalytic functionality. Mn and Fe ions can undergo reversible valence transitions (Mn²⁺/Mn³⁺ and Fe²⁺/Fe³⁺), facilitating electron hopping and accelerating charge transfer, while Zn²⁺ helps stabilize the crystal structure and modulate adsorption behavior. This synergistic combination results in enhanced sensitivity, lower detection limits, and reliable simultaneous determination of both heavy metal ions^40^. Overall, the sensing mechanism combines adsorption-assisted preconcentration, efficient electrodeposition, catalytic stripping oxidation, and rapid charge transport, which collectively explain the superior analytical performance of the MnZnFe NPs/CPE sensor.

### Individual detection of Cd²^+^ and Pb²^+^ at MnZnFe NPs/CPE

**Fig. 9:**
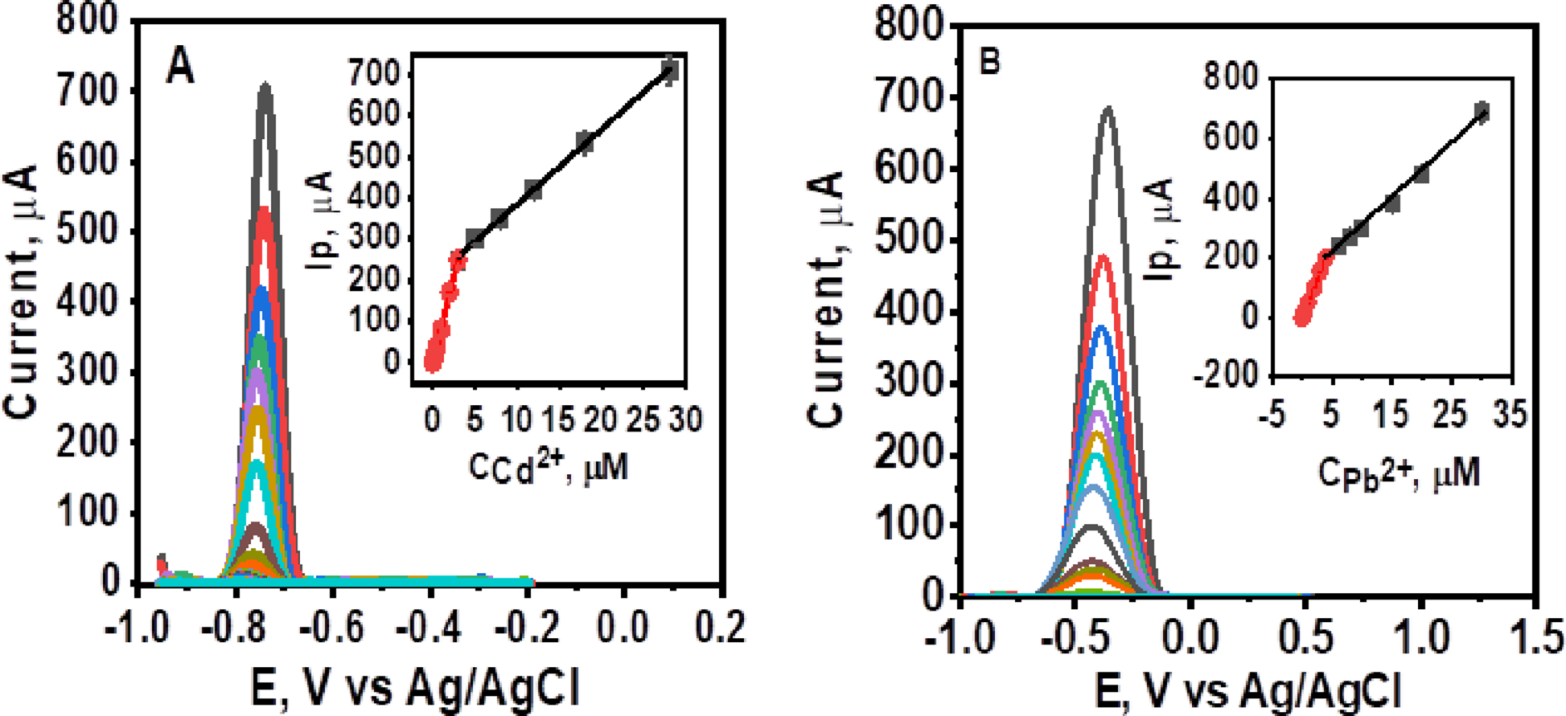
ASDPV response in 0.1 M acetate buffer (pH 5.0) with increasing concentrations of Cd²^+^ from 0.8 nM to 28.0 µM (A) and Pb²^+^ from 0.12 nM to 30.0 µM (B) using the MnZnFe NPs/CPE, at deposition potential = −1.2 V, deposition time = 120 s, pulse height 40 mV, pulse width 0.05 s, step high 20 mV and step width 0.1 s. Insets display the corresponding calibration curves.

Under optimized conditions, the target metal ions were detected using ASDPV with the MnZnFe NPs/CPE sensor. Figure 9A, B shows the voltammetric response to increasing concentrations of Cd²^+^ (0.8 nM – 28.0 µM) and Pb²^+^ (0.12 nM – 30.0 µM) in 0.1 M acetate buffer (pH 5.0). Two distinct linear calibration ranges were identified for each metal (**insets**, Fig. 9A, B), (0.80 nM – 2.0 µM and from 2.0 µM – 28.0 µM) for Cd²^+^ and (0.12 nM – 4.0 µM and 4.0 µM – 30.0 µM) for Pb²^+^. The corresponding calibration equations are:

For Cd²^+^:

Low range: Ip (µA) = 90.7 C_Cd_^2+^ (µM) + 1.95 (R² = 0.998).

High range: Ip (µA) = 18.19 C_Cd_^2+^ (µM) + 203.21 (R² = 0.999).

For Pb²^+^:

Low range: Ip (µA) = 50.30 C_Pb_^2+^ (µM) + 0.50 (R² = 0.999).

High range: Ip (µA) = 18.71 C_Pb_^2+^ (µM) + 115.70 (R² = 0.995).

The steeper slope in the lower concentration range indicates higher sensitivity, attributed to the facile oxidation of sparse metal ions on the electrode surface. Conversely, the shallower slope at higher concentrations likely results from slower oxidation kinetics and possible surface contamination by oxidation products. The limits of detection (LOD) and quantification (LOQ), calculated as 3SD/S and 10SD/S respectively (where SD is the standard deviation of the peak current for the lowest concentration, *n* = 3, and S is the slope of the corresponding low-concentration calibration curve^41^ were 0.21 nM and 0.71 nM for Cd²^+^, and 0.03 nM and 0.10 nM for Pb²^+^. These results confirm the sensor’s suitability for individual ion determination.

In this work, the electrochemical detection of Cd^2+^ and Pb^2+^ using MnZnFe NPs/CPE electrode is compared with other material-modified electrodes, as summarized in Table 1. The developed sensor exhibits superior sensitivity and a lower detection limit for the voltammetric detection of both Cd^2+^ and Pb^2+^ ions. Therefore, MnZnFe NPs represent a highly promising electrode material for the sensitive and selective determination of toxic heavy metal ions in environmental and biological samples.

**Table 1:** Comparison of the developed MnZnFe NPs/CPE sensor with previously reported modified electrodes for Cd^2+^ and Pb^2+^ detection.

Electrodes	Technique	Linear range (nM)	LOD (nM)		Ref
			Cd^2+^	Pb^2+^	
**Nafion@MWCNTs** **@PEG-Fe**_**3**_ **O**_**4**_ **/BiF/GCE** ^**a**^	**ASDPV**	**0.002–0.5**	**0.49**	**0.46**	^18^
**CoFe**_**2**_ **O**_**4**_ **@CTS/GCE** ^**b**^	**AdSDPV**	**22.24–273.1 (Cd²⁺)** **12.07–148.17 (Pb²⁺)**	**2.76**	**0.19**	^42^
**PDA@MWCNTs/GCE** ^**c**^	**ASDPV**	**10–7000**	**0.21**	**0.13**	^43^
**BHAHS@NC/MnO**_**2**_ **/CPE** ^**d**^	**DPV**	**2000–28,000 (Cd²⁺)** **1500–9100 (Pb²⁺)**	**128**	**32**	^44^
**MWCNs-COOH-PATP-AgNPs/GCE** ^**e**^	**ASSWV**	**8–50 (Cd** ^**2+**^ **)** **0.5–60 (Pb** ^**2+**^ **)**	**1.47**	**0.125**	^45^
**TPT-COF/GCE** ^**f**^	**ASSWV**	**5–300 (Cd** ^**2+**^ **)** **3–130 (Pb** ^**2+**^ **)**	**1.8**	**1.1**	^46^
**Fe**_**3**_ **O**_**4**_ **-CNF/SPE** ^**g**^	**ASSWV**	**6000–80,000 (Cd** ^**2+**^ **)** **14,000–80,000 (Pb** ^**2+**^ **)**	**61.5**	**15.4**	^47^
**Ag@COF/GCE** ^**h**^	**ASSWV**	**30–300 (Cd** ^**2+**^ **)** **20–120 (Pb** ^**2+**^ **)**	**11.1**	**6.9**	^48^
**MnZnFe NPs/CPE**	**ASDPV**	**0.80–28,000 (Cd** ^**2+**^ **)** **0.12–30,000 (Pb** ^**2+**^ **)**	**0.21**	**0.03**	Our work

^a^Nafion polymer and polyethylene glycol incorporated Fe_3_O_4_ magnetic nanoparticles (PEG-Fe_3_O_4_) and multi-walled carbon nanotubes (MWCNTs) electrochemically deposited with bismuth film modified glassy carbon electrode, ^b^small-size chitosan cobalt ferrite core−shell nanoparticles modified glassy carbon electrode, ^c^polydopamine-functionalized multi-walled carbon nanotubes modified glassy carbon electrode, ^d^(*E*)−4-((5-bromo-2-hydroxybenzylidene) amino)−3-hydroxynaphthalene-1-sulfonic acid) (5-BHAHS), onto nano-cellulose NC along with MnO_2_ nanoparticles modified carbon paste electrode, ^e^multiwall carbon nanotubes-Poly(2-aminothiophenol) @silver nanocomposite modified glassy carbon electrode, ^f^2,4,6-triaryloxy-1,3,5-triazine based covalent organic framework modified glassy carbon electrode, ^g^carbon nanofibers and Fe_3_O_4_ nanoparticles modified screen-printed electrode, ^h^silver nanoparticle-embedded covalent organic framework modified glassy carbon electrode.

In addition to the analytical performance comparison presented in Table 1, a qualitative assessment of the fabrication cost and complexity of the reported electrodes is also important for evaluating their practical applicability. As summarized, MWCNTs/GCE-based systems typically involve carbon nanotubes, polymers, and metal nanoparticles, requiring multi-step modification procedures and resulting in relatively high cost. Similarly, COF/GCE systems rely on covalent organic frameworks and other functional nanomaterials synthesized through complex routes, which further increases their cost. AgNPs/GCE-based electrodes, although highly sensitive, depend on noble metal nanoparticles and moderately complex fabrication steps, also contributing to high overall cost. In contrast, screen-printed electrode (SPE)-based systems benefit from commercial fabrication and portability but still involve moderate cost due to manufacturing and material requirements. Previously reported CPEs offer a simpler and more economical alternative, with low to moderate costs depending on the modifier used. Notably, the developed MnZnFe NPs/CPE in this work employs inexpensive and readily available materials (graphite powder, paraffin oil, and ferrite nanoparticles) and is fabricated through a simple in-laboratory procedure without the need for sophisticated equipment or costly reagents. Therefore, it represents a low-cost, yet efficient sensing platform compared to the other electrodes listed in Table 1, making it particularly suitable for routine and on-site environmental monitoring applications.

### **Simultaneous detection of Cd²**^**+**^**and Pb²**^**+**^**at MnZnFe NPs/CPE**.

**Fig. 10:**
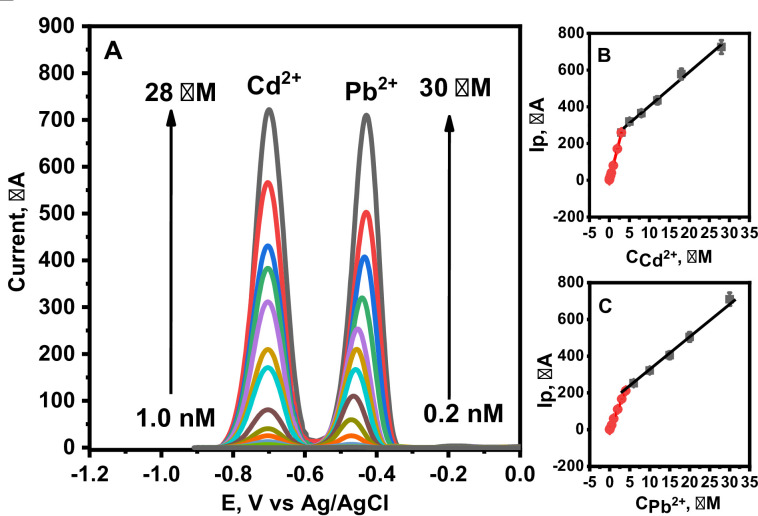
(**A**) ASDPVs of MnZnFe NPs/CPE in 0.1 M acetate buffer (pH 5.0) contain various concentrations of Cd^2+^ (1.0 nM − 28.0 µM), and Pb^2+^ (0.2 nM − 30.0 µM). at deposition potential = −1.2 V, deposition time = 120 s, pulse height 40 mV, pulse width 0.05 s, step high 20 mV and step width 0.1 s. (**B**), and (**C**) display the corresponding calibration curves.

A key advantage of the fabricated sensor is its ability to simultaneously distinguish and quantify target heavy metals in a mixture. Figure 10A shows a series of single ASDPV scans using the MnZnFe NPs/CPE for mixed solutions containing increasing concentrations of Cd²^+^ (8.0 nM – 25.0 µM) and Pb²^+^ (3.0 nM – 23.0 µM). Well-defined, separate oxidation peaks for both metals are visible, with their currents increasing proportionally with concentration. The linear relationship between peak current and concentration for each ion is detailed in Fig. 10B, C revealing two distinct calibration ranges per analyte, (0.80 nM – 2.0 µM and 2.0 µM – 28.0 µM) for Cd^2+^ and (0.12 nM – 4.0 µM and 4.0 µM – 30.0 µM) for Pb^2+^. The corresponding calibration equations are:

For Cd²^+^:

Low range: Ip (µA) = 85.33 C_Cd_^2+^ (µM) + 1.10 (R² = 0.998).

High range: Ip (µA) = 18.6 C_Cd_^2+^ (µM) + 217.7 (R² = 0.999).

For Pb²^+^:

Low range: Ip (µA) = 53.6 C_Pb_^2+^ (µM) + 1.85 (R² = 0.999).

High range: Ip (µA) = 19.1 C_Pb_^2+^ (µM) + 129.7 (R² = 0.995).

From these calibrations, the LODs were calculated as 0.27 nM for Cd²^+^ and 0.045 nM for Pb²^+^. This excellent sensitivity confirms the sensor’s capability for the simultaneous determination of both ions. The performance is attributed to the superior electrocatalytic properties and synergistic effects of Mn–Zn ferrites, establishing it as an efficient platform for multi-metal detection.

### Interference study

To assess the selectivity and practical viability of the developed sensor, an interference study was conducted for the simultaneous detection of Cd²^+^ and Pb²^+^. The influence of potentially coexisting ions including Zn²^+^, Cu²^+^, Fe³^+^, Ni²^+^, Co²^+^, Mg²^+^, Ca²^+^, K^+^ and Na^+^ was evaluated at concentrations typical of environmental samples. Each interferent was tested at a level at least 100-fold higher than the target analytes **Fig S4**. ASDPV measurements revealed that the MnZnFe NPs/CPE produced well-defined, distinct stripping peaks for Cd²^+^ and Pb²^+^ with minimal signal overlap or current variation. While Zn²^+^ and Cu²^+^ caused slight interference due to comparable redox potentials, this effect was negligible under the optimized experimental conditions. Other common ions (Fe³^+^, Ni²^+^, Co²^+^, Na^+^, Mg²^+^, and Ca²^+^) showed no significant impact.

**Table ****S1** involves detailed percentage changes in current, complete with standard deviation bars for all tested substances, which confirms this minimal interference. The results confirm the sensor’s excellent selectivity, demonstrating its capability to accurately quantify Cd²^+^ and Pb²^+^ in complex matrices and underscoring its suitability for real-world environmental water analysis.

### Repeatability, reproducibility and stability of MnZnFe NPs/CPE

The repeatability, reproducibility, and stability of the MnZnFe NPs/CPE were evaluated to confirm its reliability for detecting Cd²^+^ and Pb²^+^. Repeatability was assessed via six consecutive ASDPV measurements of 15.0 µM solutions using the same electrode (**Fig. S5A**,** D**), yields low relative standard deviations (RSDs) of 1.4% for Cd²^+^ and 1.9% for Pb²^+^. Reproducibility tests across six independently fabricated electrodes (**Fig. S5B**,** E**) showed even lower RSDs of 0.42% (Cd²^+^) and 1.39% (Pb²^+^), confirms excellent manufacturing consistency. Long-term stability was also strong; after 30 days of dry storage at room temperature; the sensor retained over 94.1% and 93.6% of its initial response for Cd²^+^ and Pb²^+^, respectively as indicated in (**Fig S5C**,** F**). These results demonstrate the robustness of the MnZnFe NPs/CPE, making it suitable for routine and on-site environmental analysis.

### Real sample application

To assess the real-world utility and reliability of the developed MnZnFe NPs/CPE electrochemical sensor, its performance was evaluated by analyzing real environmental water samples for Cd²^+^ and Pb²^+^ content.

Samples were collected from representative local sources, including tap water and Nile River water. Each sample was prepared by filtration to remove suspended particulates, followed by acidification to pH ~ 5.0 using an acetate buffer solution. This step both optimized the analytical conditions and helped preserve the ionic state of the target metals. To ensure accuracy and account for potential matrix interference, quantification was performed using the standard addition method. Known concentrations of Cd²^+^ and Pb²^+^ standards were introduced into the prepared samples. The recovery values, which indicate the accuracy of the measured concentration versus the known spiked amount, were calculated to be within the range of 94.0% to 104.0% (Table 2). Accompanying relative standard deviations (RSDs) were all below 5%, confirming the high precision of the measurements. The excellent recovery rates and low RSDs demonstrate that the sensor delivers accurate and reproducible results even in complex environmental matrices. This successful application confirms the practical viability of the modified electrode for the direct and reliable detection of trace heavy metal ions in real water samples, underscoring its potential for environmental monitoring and on-site analysis.

**Table 2:** The analysis results of Cd^2+^ and Pb^2+^ in real water samples at MnZnFe NPs/CPE (*n* = 3).

Sample	Analyte	Added (µM)	Found (µM)	Recovery (%)	RSD%
Nile River water	**Cd** ^**2+**^	1.00	1.04	104.00	1.30
		5.00	4.96	102.30	2.04
		15.00	14.48	96.53	0.82
	**Pb** ^**2+**^	1.00	0.94	94.00	1.02
		5.00	4.92	98.40	0.96
		15.00	15.09	104.90	1.60
Tap water	**Cd** ^**2+**^	1.00	0.99	99.00	1.05
		5.00	5.02	100.40	1.90
		15.00	14.92	99.47	0.78
	**Pb** ^**2+**^	1.00	0.98	98.00	1.13
		5.00	4.95	99.00	0.99
		15.00	14.98	99.86	1.43

## Conclusion

In this study, a novel, sensitive, and reliable electrochemical sensor was successfully developed for the simultaneous determination of trace levels of Cd²^+^ and Pb²^+^ ions. The sensor was fabricated by modifying a conventional carbon paste electrode with spinel MnZnFe NPs (MnZnFe NPs/CPE), synthesized via a facile co-precipitation method. Comprehensive characterization through FTIR, XRD, and SEM&EDX confirmed the successful formation of the nanospinel ferrite with the intended composition and a favorable nanostructured morphology, which provided a high surface area and abundant active sites.

The MnZnFe NPs/CPE demonstrated superior electrochemical performance compared to the bare CPE. The synergistic combination of Mn and Zn within the ferrite lattice was crucial to its function: the redox-active manganese centers facilitated efficient electron transfer, while the zinc ions contributed to enhanced adsorption and preconcentration of the target metal ions. Under optimized conditions, the modified electrode exhibited well-defined, sharp, and significantly enhanced stripping peaks for both Cd²^+^ and Pb²^+^, with excellent peak separation, enabling their simultaneous quantification without mutual interference. The sensor provided exceptional analytical figures of merit. It showed wide linear dynamic ranges for both ions, with remarkably low limits of detection (LOD) reaching nanomolar concentrations, which are well below the WHO permissible limits for drinking water. The modified electrode also displayed good repeatability, reproducibility, and acceptable long-term stability. Furthermore, the sensor exhibited high selectivity in the presence of common interfering ions and was successfully applied to the analysis of Cd²^+^ and Pb²^+^ in real water samples (tap and river water), with satisfactory recovery rates, validating its practical utility for environmental monitoring.

## Supplementary Information

Below is the link to the electronic supplementary material.

Supplementary Material 1

Supplementary Material 2

## Data Availability

All data generated or analyzed during this study are included in this published article [and its supplementary information files].
