# Supplementary material for: Highly sensitive voltammetric determination of Pb²⁺ and Cd²⁺ ions using a carbon paste electrode modified with Mn₀.₅Zn₀.₅Fe₂O₄ spinel ferrite nanoparticles
Source: Sci Rep. 2026 May 20;16:15756. doi: 10.1038/s41598-026-52676-4 (PMC13190725; doi:10.1038/s41598-026-52676-4)
Supplement: Supplementary file 1 — Supplementary Material 1 [file 41598_2026_52676_MOESM1_ESM.docx]

**Highly Sensitive Voltammetric Determination of Pb²⁺ and Cd²⁺ Ions Using a Carbon Paste Electrode Modified with Mn₀.₅Zn₀.₅Fe₂O₄ Spinel Ferrite Nanoparticles**

Asmaa A. Khodari^a*+^, Ahmed A. Shamroukh^a*+^, Ahmed R Tawfik^a+^, Hassan M. A. Salman^a^, Hytham.F. Assaf^a^

^a^Chemistry Department, Faculty of Science, Qena University, Qena, 83523, Egypt.

+ These authors contributed equally: Asmaa A. Khodari, Ahmed. A. Shamroukh, Ahmed R. Tawfik

*E-Mail: [atawfik2018@gmail.com](mailto:atawfik2018@gmail.com); [ahmed.shamrouk@sci.svu.edu.eg](mailto:ahmed.shamrouk@sci.svu.edu.eg)

**Supplementary Material**

**EDX analysis**

**
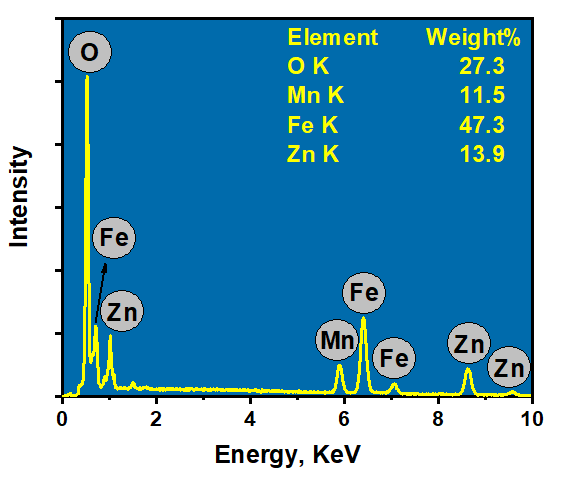

Fig. S1** EDX spectrum of MnZnFe NPs.

**Optimization of the amount of modifier in the electrode**

The proportion of the MnZnFe NPs within the carbon paste was a critical factor for sensor performance. As shown in **Fig. S2**, the anodic peak current for both metals intensified with modifier content up to an optimum of 15% (w/w), beyond which the signal stabilized and then diminished. The initial enhancement is attributed to a greater number of active sites for both metal ions adsorption, while the subsequent decline is likely due to increased electrical resistance from excessive non-conductive modifier. Consequently, a 15% (w/w) load was selected for all subsequent experiments.

**Fig. S2** Influence of MnZnFe NPs content in the carbon paste on the electrode response.

**Effect of deposition potential and deposition time**

**Fig. S3** Effect of deposition potential (**A**) and deposition time (**B**) on the ASDPV response currents of Cd^2+^ (1.0 µM) and Pb^2+^ (2.0 µM) in 0.1 M acetate buffer (pH 5.0) at MnZnFe NPs/CPE.

**Interference Study**


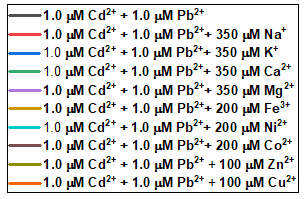


**Fig. S4** The ASDPV curves of 1.0 μM Cd^2+^ and 1.0 μM Pb^2+^ at MnZnFe NPs/CPE in the presence of possible interfering ions. Deposition potential: -1.2 V, deposition time: 120 s.
**Table S1** The detailed percentage changes in current (i/I_0_​) of 1.0 μM Cd^2+^ and 1.0 μM Pb^2+^ in the presence of possible interfering ions at MnZnFe NPs/CPE in presence of various interferents.

| Interference | i/I_0_​ | RSD |
| --- | --- | --- |
| Na^+^ | 1.02 | 0.63 |
| K^+^ | 1.09 | 0.31 |
| Ca^2+^ | 1.032 | 0.81 |
| Mg^2+^ | 1.032 | 0.72 |
| Fe^3+^ | 1.02 | 0.35 |
| Ni^2+^ | 0.98 | 0.37 |
| Co^2+^ | 1.11 | 0.38 |
| Cu^2+^ | 0.98 | 0.43 |
| Zn^2+^ | 1.09 | 0.40 |

**Repeatability, reproducibility and stability of MnZnFe NPs/ CPE**

**Fig. S5** ASDSVs of 15.0 µM Cd^2+^ and 15.0 µM Pb^2+^ in 0.1 M acetate buffer (pH 5.0) on the MnZnFe NPs/CPE repeatability (A and D), reproducibility (B and E), and stability (C and F) studies.
